# Algorithm-based decision support for symptom self-management among adults with Cancer: results of usability testing

**DOI:** 10.1186/s12911-018-0608-8

**Published:** 2018-05-29

**Authors:** Mary E. Cooley, Janet L. Abrahm, Donna L. Berry, Michael S. Rabin, Ilana M. Braun, Joanna Paladino, Manan M. Nayak, David F. Lobach

**Affiliations:** 1 The Phyllis F. Cantor Center, Dana-Farber Cancer Institute, 450 Brookline Avenue, Boston, MA 02115 USA; 2 Department of Psychosocial Oncology and Palliative Care, Dana-Farber Cancer Institute, 450 Brookline Avenue, Boston, MA 02115 USA; 3 The Phyllis F. Cantor Center and the Department of Medicine, Dana-Farber Cancer Institute, 450 Brookline Ave, LW-512, Boston, MA 02115 USA; 4 Lowe Center for Thoracic Oncology, Dana-Farber Cancer Institute, 450 Brookline Avenue, Boston, MA 02115 USA; 5 Klesis Healthcare and Department of Family Medicine, Durham, NC 27705 USA; 6 Department of Family Medicine, Duke University Medical Center, 2100 Erwin Road, Durham, NC 27710 USA

**Keywords:** Rule-based clinical decision support, Symptom management, Patient engagement, Patient self-management, Cancer

## Abstract

**Background:**

It is essential that cancer patients understand anticipated symptoms, how to self-manage these symptoms, and when to call their clinicians. However, patients are often ill-prepared to manage symptoms at home. Clinical decision support (CDS) is a potentially innovative way to provide information to patients where and when they need it. The purpose of this project was to design and evaluate a simulated model of an algorithm-based CDS program for self-management of cancer symptoms.

**Methods:**

This study consisted of three phases; development of computable algorithms for self-management of cancer symptoms using a modified ADAPTE process, evaluation of a simulated model of the CDS program, and identification of design objectives and lessons learned from the evaluation of patient-centered CDS. In phase 1, algorithms for pain, constipation and nausea/vomiting were developed by an expert panel. In phase 2, we conducted usability testing of a simulated **s**ymptom **a**ssessment and **m**anagement **i**ntervention for self-care (SAMI-Self-Care) CDS program involving focus groups, interviews and surveys with cancer patients, their caregivers and clinicians. The Acceptability E-scale measured acceptability of the program. In phase 3, we developed design objectives and identified barriers to uptake of patient-centered CDS based on the data gathered from stakeholders.

**Results:**

In phase 1, algorithms were reviewed and approved through a consensus meeting and majority vote. In phase 2, 24 patients & caregivers and 13 clinicians participated in the formative evaluation. Iterative changes were made in a simulated SAMI-Self-Care CDS program. Acceptability scores were high among patients, caregivers and clinicians. In phase 3, we formulated CDS design objectives, which included: 1) ensure patient safety, 2) communicate clinical concepts effectively, 3) promote communication with clinicians, 4) support patient activation, and 5) facilitate navigation and use. We identified patient barriers and clinician concerns to using CDS for symptom self-management, which were consistent with the chronic care model, a theoretical framework used to enhance patient-clinician communication and patient self-management.

**Conclusion:**

Patient safety and tool navigation were critical features of CDS for patient self-management. Insights gleaned from this study may be used to inform the development of CDS resources for symptom self-management in patients with other chronic conditions.

## Background

Patient-centered healthcare is one of six aims to improve the United States healthcare system [1]. One facet of patient-centered care is engaging patients and encouraging self-management, especially in the context of chronic illness. Self-management programs that provide coaching and education, and are supported by timely information, facilitate patient engagement [2, 3].

Given the frequency of symptoms from cancer and its treatment, it is essential that patients (and their caregivers) understand how to self-manage symptoms and when to call their clinicians for advice. McCorkle and colleagues [3–5] noted that most patients with cancer try to self-manage their care and that developing system-level interventions to support self-management is essential for quality cancer care. Evidence-based strategies to assist patients with self-management include: education, telephone consultations, Internet tools for tracking disease-specific parameters, and coaching [3, 6–13]. Such interventions targeted information management, medications, psychological consequences of illness, lifestyle, social support, communication, accessing services, and setting goals [14–16]. Self-management interventions decreased the severity of pain [17], fatigue [18], and depression [19]. Web-based interventions, featuring self-monitoring, education and coaching of patients combined with summaries of patient-reported data delivered to clinicians, led to improvement in symptom distress and quality of life [12, 16, 20]. Evidence suggests that reporting symptoms to clinicians alone may not be sufficient. A project in which patients receiving chemotherapy reported on symptoms using an automated phone system failed to impact care. Even though symptoms of moderate to severe intensity were reported, clinicians did not provide patients with management guidance [10].

Considering the variability of clinician response to symptom reports, an optimal approach to enhance self-management includes providing patient-specific, real-time, actionable information. Supplying Clinical Decision Support *(CDS)* directly to patients may enhance their ability to self-manage symptoms. *CDS* “provides individuals with person-specific information, intelligently filtered or presented at appropriate times, to enhance health” [21]. Four studies have described rule-based CDS tools for cancer patients. [12, 20, 22, 23] In two studies, CDS was used to identify the presence of a symptom using an algorithm with a single decision node that generated general recommendations for symptom management [12, 22]. In studies by Berry and colleagues [24] and Weaver, [23] the focus was self-care support and identifying symptoms to report to clinicians. Berry and colleagues [12] found that Web-based coaching of patients to report symptom experiences verbally resulted in more frequent reports during clinic visits, but the investigators did not document how often a participant called the clinic to ask for help. Ruland et al. [20] developed a Web-based resource that assisted patients with assessing symptoms, finding information, communicating with clinic personnel and provided self-management advice. This tool was evaluated and showed a slight decrease in symptom distress for patients [20] but the CDS component was not evaluated independently for its effect. A follow-up study determined that communication with nurses was the most valued component [25].

The present study extends the literature by reporting on the design and formative evaluation of an algorithm-based simulated model of a patient-centered CDS program that facilitates cancer symptom self-management, provides advice on when patients should contact their clinicians, and includes coaching information about what to tell them. The goals of the project were to develop computable algorithms for pain, constipation and nausea/vomiting in phase 1, conduct iterative usability testing of the simulated CDS program called the **S**ymptom **A**ssessment and **M**anagement **I**ntervention for **Self-Care** (SAMI-Self-Care) with patients, their caregivers and clinicians in phase 2, and develop design objectives and identify barriers to uptake of patient-centered CDS based on the data gathered from stakeholders, which included members of an expert panel, patients, caregivers, and their clinicians in phase 3.

## Methods

We employed a convergent, parallel, mixed methods design, [26] in which we collected qualitative and quantitative data in parallel, analyzed it separately, and then merged all data during interpretation, to evaluate the acceptability of a simulated algorithm-based CDS program for cancer symptom self-management. This study was conducted at Dana-Farber Cancer Institute (DFCI) and approved by the Institutional Review Board (IRB), Protocol number-12-300.

### Phase 1: Algorithm-based CDS intervention development

In order to develop the patient-centered CDS algorithms, we used a modified ADAPTE process [27–29] consisting of five steps; 1) identify expert panels to develop and evaluate the computable algorithms, 2) develop groups to work on each symptom and synthesize the literature, 3) convene groups to translate evidence-based information into computable algorithms for each symptom, 4) conduct peer review on the content of the algorithms before convening a multidisciplinary consensus meeting, and 5) hold a multidisciplinary panel meeting to review, modify and approve the algorithms.

The study team identified areas of expertise that were critical for developing and evaluating an algorithm-based CDS tool for symptom self-management. Individuals with the desired expertise were identified through existing professional relationships or through recommendations of colleagues. The identified individuals were invited to join the panel by the principal investigator (MEC) and included: stakeholders with expertise in clinical care (oncologists, palliative care experts, psychiatrists, and oncology nurses), information systems (experts in CDS, patient data collection, health communication, and graphic design), care delivery process (experts in workflow, quality improvement, and health equity), as well as patients and caregivers.

Our expert panel drew from evidence-based resources and worked with CDS experts to develop computable algorithms that would enable self-management of pain, constipation, and nausea/vomiting. Overall, the expert panel met four times for 4 h each time to review the algorithms developed by the research team. The symptoms chosen were the most common reasons for urgent care among cancer patients and identified as important targets for symptom management by patients and their caregivers in a previous study that explored patient preferences for CDS to enhance clinical care [30]. Our process of developing algorithms was based on our previous work that adapted evidence-based guidelines for clinicians [31]. Similar to our previous work, multi-disciplinary groups were formed for each symptom and serial meetings were held to develop the initial algorithms. Once the algorithms were completed, a group meeting with the members of the research team and the expert panel was held to review, modify and approve the algorithms. For each symptom, characteristics were identified to direct self-management. Characteristics of pain included descriptors of neuropathic or somatic pain, and a measure of intensity (moderate, severe). Features of nausea/vomiting included chemotherapy-induced, radiation-induced, chemoradiation-induced, acid reflux, vertigo, recent narcotic increase, anticipation of chemotherapy, and constipation. Characteristics of constipation included frequency and consistency of stool.

### Phase 2: Usability testing

In order to refine the algorithm content and the simulated SAMI-Self-Care tool display and function iteratively, we used a collaborative-participatory approach. This approach engaged stakeholders in a shared co-designing process and helped ensure that the product will meet real-life needs and be adopted [32, 33]. The team formulated a scenario for a symptom management dilemma that would allow patient users to traverse the self-care management algorithms so that self-management recommendations could be generated. Cognitive testing was performed for each algorithm by reviewing the content displayed on an iPad with patients and their caregivers to ensure that these questions were clear, understandable, and relevant, and that the response options were appropriate prior to initiating the iterative usability testing. Cognitive testing of items is recommended as a standard approach to the development process as it allows one to ascertain whether participants understand items on a questionnaire and allows for iterative changes before finalizing questions [34].

This patient-vetted algorithm content was given to the Dana-Farber Harvard Comprehensive Cancer Center Health Communication Core. The Core developed a mockup of the SAMI-Self-Care using interactive PDF files to simulate the function of the CDS tool based on expert panel input and clinical informatics literature [35, 36]. The PDF files imitated the functionality of a Website and allowed participants to experience the “look and feel” of a potential operational CDS system on an iPad. This approach provided a practical and economical approach to usability testing, especially during formative stages of development when iterative refinement is necessary. The Health Communication Core also designed reports containing self-management recommendations to elicit feedback from participants regarding report content and appearance. A series of focus groups or interviews were conducted to elicit feedback about the CDS program. Using a multi-methods approach (i.e. focus groups and interviews) is appropriate when the intent is to gather information that is focused on specific goals and questions, which in this case was participant response about the usability of an algorithm-based CDS program [37]. We used a combination of methods to provide flexibility to participants to be able to participate in the usability sessions. We used the same facilitator to conduct the focus groups and interviews and used an interview guide **t**o elicit information about the usability and acceptability of the SAMI-Self-Care CDS program.

**Fig. 1:**
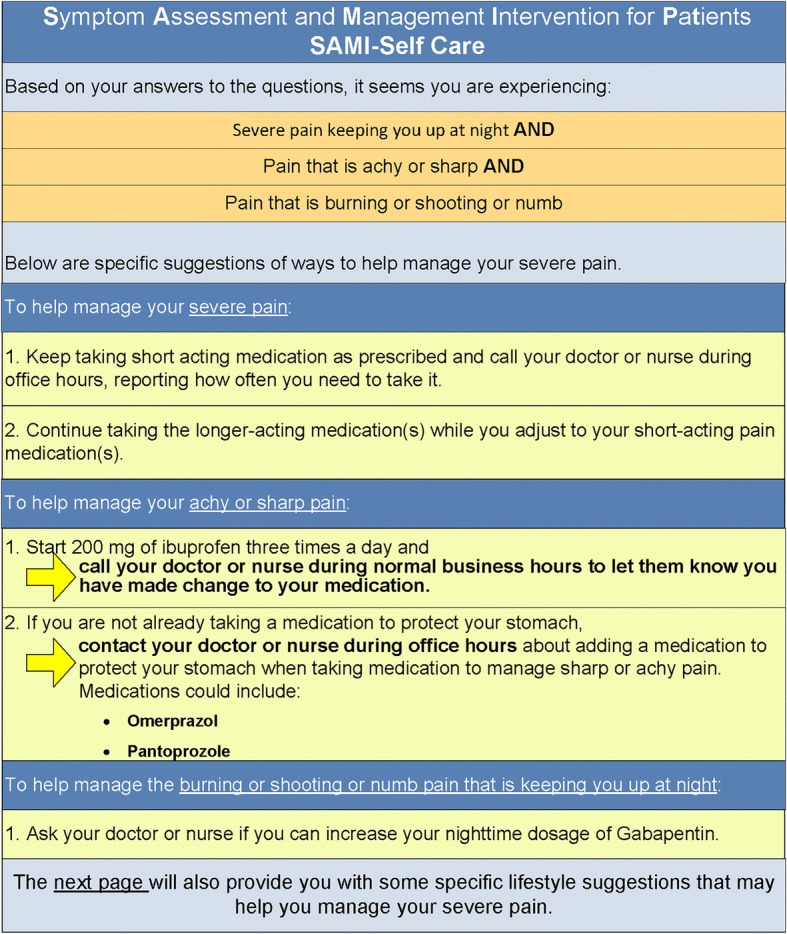
Initial SAMI-Self Care report

**Fig. 2:**
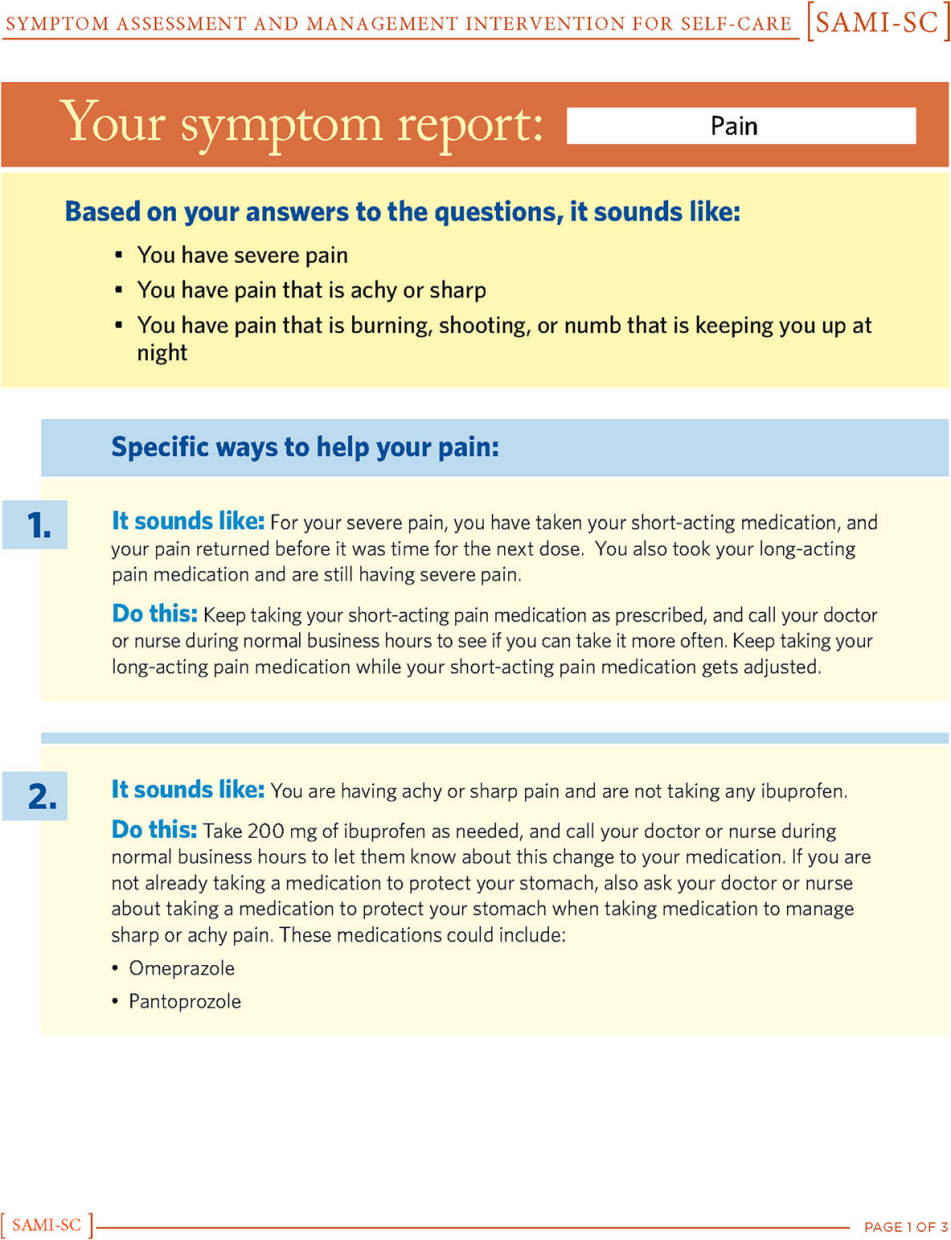
First revision for SAMI-Self Care report

**Fig. 3:**
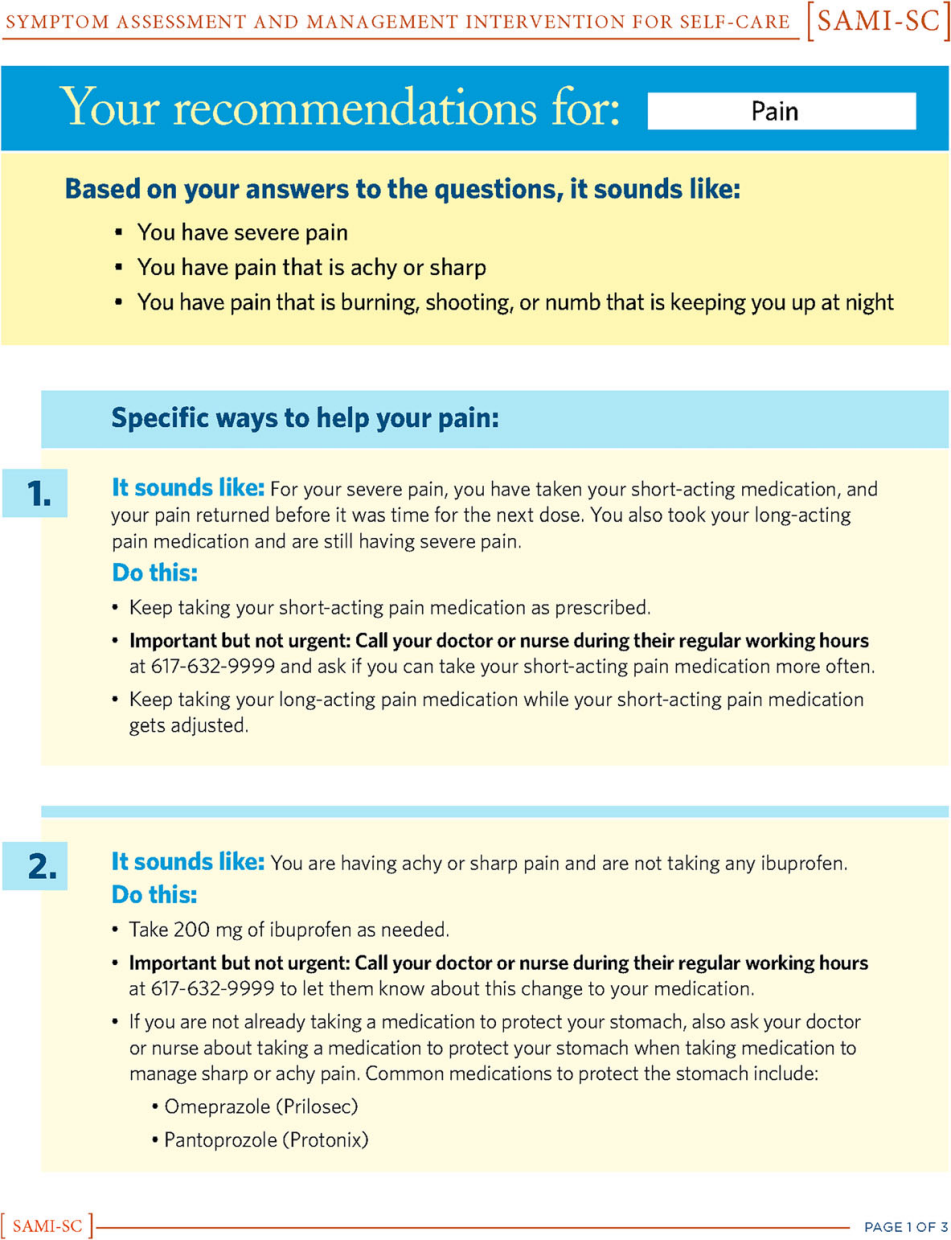
Second revision for SAMI-Self Care report

**Fig. 4:**
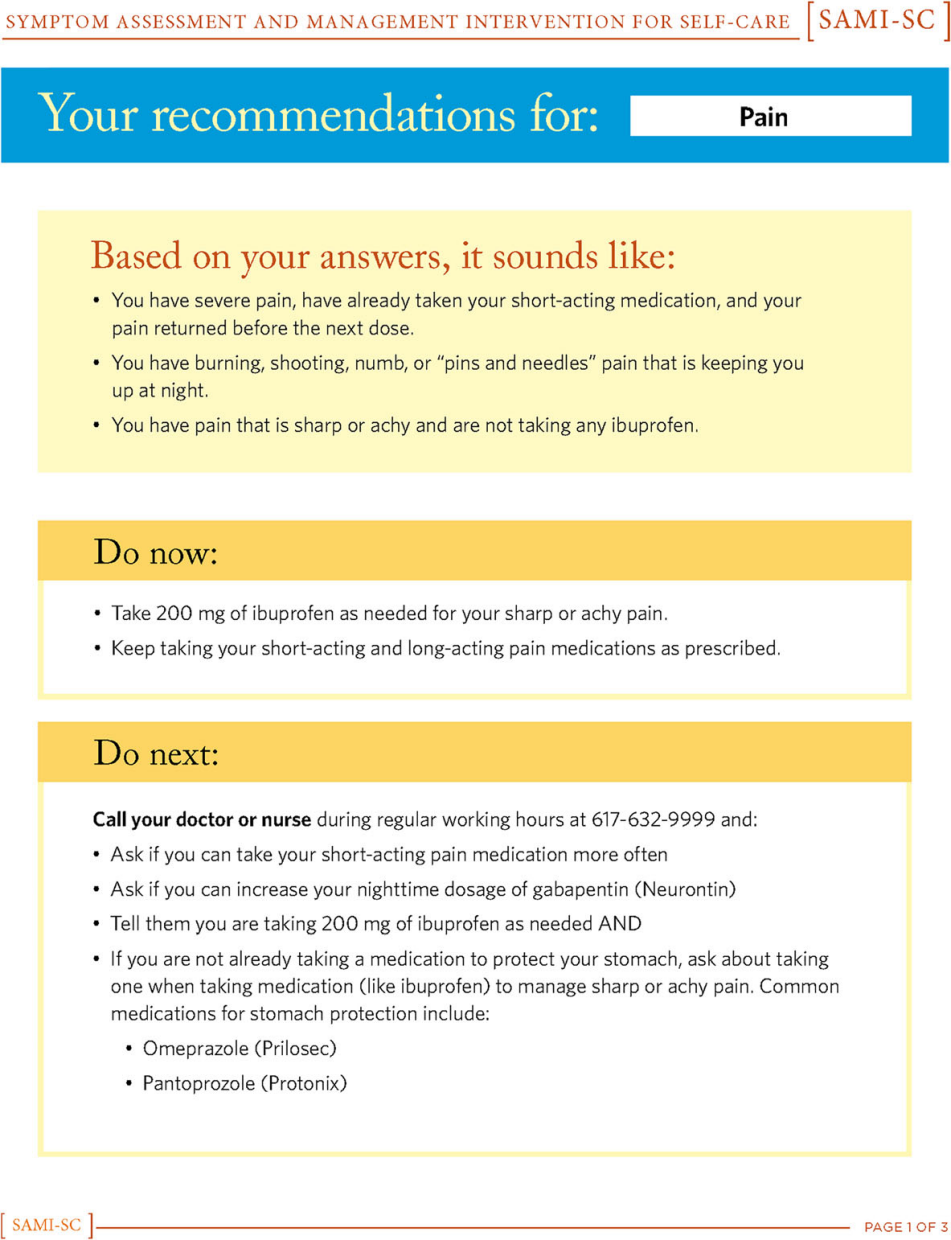
Third revision for SAMI-Self Care report

Based on feedback from the expert panel, patients, caregivers, and clinicians, we iteratively created three successive versions of the SAMI-Self-Care program. Figures 1, 2, 3, 4 show the iterative development of the self-management report that was generated for participants based on responses to the questions and algorithm-based CDS. Feedback from the usability testing was used to inform the changes that were made to the CDS program and the self-management report (see results).

### Participant recruitment

Patients were identified through medical records at DFCI under a waiver of consent authorization, and names were submitted to their clinicians for approval to contact patients, as required by the IRB. Eligible patients were age ≥ 18, English speaking, and had received cancer treatment within the past 6-months. All approved patients were sent an initial letter describing the study and inviting them to participate. Patients received two follow-up calls if they didn’t respond to the letter. Patients identified caregivers to invite to participate in the study. Eligible caregivers were age ≥ 18 years and provided care to a patient who received cancer treatment within the past 6-months. Deliberate efforts were made to sample across a range of diagnoses, educational backgrounds, ages, genders, and races. All participants signed consent forms at the time of participation. Each participant received $50 for completing the study.

Clinicians in ambulatory oncology included physicians, nurse practitioners, physician assistants, and registered nurses. Clinicians were identified through clinic rosters. An invitation to participate in the study was emailed to eligible clinicians**.** Two follow-up emails were sent if no response was received. Clinicians were recruited for either focus groups or interviews depending on their schedules. Clinicians received an invitation letter describing the study but signed consent was waived by the IRB. Each clinician received $100 for completing the study.

### Focus group and interview processes

Focus groups and interviews were held separately for clinicians and patients/caregivers. Focus groups included up to five participants; interviews included one or two participants and were held in a convenient conference room at DFCI. Meetings for clinicians lasted about 30 min whereas those for patients and caregivers lasted 45–60 min. One symptom was addressed per session. Using a script, the facilitator queried patients, caregivers and clinicians about the algorithm content of the SAMI-SC programs for constipation, pain, and nausea/vomiting, and asked about the “look and feel” of the materials for pain and nausea/vomiting. The topics for patients and caregivers focused on the: 1) visual appeal, 2) format and navigation, 3) understandability of written content and terminology, and 4) wording of self-management suggestions. The topics for the clinicians focused on 1) a review of the patient self-management algorithms, 2) review of the simulated SAMI-Self-Care CDS program, and 3) feedback about ways to improve the algorithms and/or simulated model. Patients/caregivers and clinicians reported demographic data using a standardized survey.

The number of sessions per symptom was determined by evidence of content saturation and absence of new common responses [38]. With patients/caregivers, we conducted six sessions for pain, seven sessions for nausea/vomiting, and three sessions for constipation. With clinicians, we conducted two sessions for pain, three sessions for nausea/vomiting, and one session for constipation. All discussions were audio-recorded and transcribed.

### Survey instruments

The Acceptability E-scale measured the acceptability of the CDS program [39]. This scale has been used in adult patients with various types of cancers from medical oncology, radiation oncology and stem cell transplant to measure acceptability of Web-based systems, including computerized symptom and quality of life assessments, patient educational materials and patient-centered decision support. Psychometric testing was conducted in 627 adults with cancer [40, 41]. Cronbach alpha reliability coefficient was 0.75 and factor analysis revealed that the scale was unidimensional [39]. Five-point Likert-type items (1–5; larger number equals higher acceptability and a score of 3 indicates a neutral response) were presented to elicit feedback from both patient/caregiver and clinician participants regarding: 1) understandability of language, 2) helpfulness of suggestions provided for symptom self-management, 3) usefulness of reports, and 4) overall satisfaction with the program. The a priori target for acceptability of SAMI-Self-Care was a mean score of ≥4 on each item since this score indicated a positive acceptability response. In addition to the Acceptability E-scale, participants were asked what other types of information would have been helpful for symptom self-management. The scale took less than 2 min to complete and was written at a 5th grade reading level.

### Analysis

Patient/caregiver and clinician demographic data were summarized as descriptive statistics**.** Acceptability E-scale results were summarized as item means and standard deviations.

Audio-recorded session content was transcribed and reviewed by three study team members (MEC, DFL, MMN), coded inductively, and grouped into common responses to identify revisions that needed to be made in the CDS tool. Common responses were then combined and reconciled though discussion. A list of suggested revisions to improve SAMI-Self-Care algorithms and the simulated model was created and ranked based on audio files and notes. Critical revisions were implemented immediately, while other less critical revisions were monitored for repetition. This process was repeated, after each iteration of CDS tool development, until no new responses or suggestions for changes emerged from the usability testing. Phase 2 analyses focused on the revisions that needed to be make to the CDS tool.

### Phase 3: Design objectives and barriers to uptake of patient-centered CDS

Once all the data were collected and the usability testing completed, three investigators (MEC, DFL, MMN) reviewed all of the qualitative data in order to identify design objectives and barriers to uptake of the patient-centered CDS program. Eight sessions were needed to organize the data collected from the expert panel, patients, caregivers and clinicians and discuss the themes that were identified through inductive content analysis [42]. These data were then shared and discussed with other members of the research team to generate a final list of design objectives, patient barriers, and clinician concerns to use of the CDS program.

### Analyses

Audio-recorded session content was transcribed and reviewed by three study team members (MEC, DFL, MMN), coded, and grouped into themes. Themes were then combined and reconciled though discussion. Inductive analyses of the coded qualitative data for phase 3 focused on interpreting patient barriers to using the CDS program, defining clinician concerns about use of CDS by patients/caregivers, and identifying strategies to overcome the areas of concern. It was noted during the analysis that the themes that emerged from the qualitative data were consistent with the Chronic Care Model [3, 43]. Thus, five design objectives detailed below (in Results) were developed to address these concerns. As a result, content analysis was then used to regroup the themes to fit within the Chronic Care Model.

## Results

### Phase 1: Rule-based CDS intervention development

Nine potential participants were approached for membership in the expert panel, and all accepted the invitation. Altogether, 16 members of the research team and expert panel contributed to algorithm development. Work sessions were conducted in person and by Web conferencing so that the flow charts could be displayed more easily for discussion. Branching logic was used to develop the algorithms and these were displayed as flow charts that were shared among all team members. Research staff provided support to access current guidelines that were available for the targeted symptoms and to assist with literature reviews. Each work group had someone assigned who was expert in pharmacological, non-pharmacological and self-management approaches so that a comprehensive approach to enable self-management would be embedded into the algorithms.

Once the development of the algorithms and usability testing was completed, a multidisciplinary consensus meeting was held with the expert panel, research team and co-investigators to review, modify and approve the final versions. In order to iteratively improve the program, the expert panel focused on several issues. The primary issue was patient safety. Safety was addressed by identifying potential serious causes of each symptom and directing patients to seek contact with their clinicians. Accordingly, all three algorithms began with the identification of “red flag symptoms” that caused patients a forced exit of the CDS tool and a directive to call a clinician immediately for guidance. A second issue was how to make the tool accessible to patients across a range of health literacies. Our solution was to provide information that patients could elect to review or skip based on their needs. A third issue was how to provide medication-related advice that was aligned with therapies recommended by a patient’s clinician. To address this issue, we asked the patient whether the medication we were recommending for the problem (e.g. senna for constipation) was approved by his/her clinician. If it was approved, we inquired whether the patient had taken that medication. If not, the patient was advised to take it. If the patient had not been prescribed the recommended therapy, he or she was advised to contact his/her clinician and inquire if this therapy could be appropriate. The resulting algorithms had a large number of decision nodes ranging from 51 to 257 (moderate and severe pain combined). A decision node is a point in the algorithm where the logic branches into two or more directions. A higher number of decision nodes reflect greater algorithmic complexity [44]. Table 1 provides information about the number of decision nodes and red flag questions that were present within each symptom algorithm. A simple majority vote was taken at the end of the discussion surrounding each symptom algorithm to determine whether agreement was reached regarding modifications and approval. Table 1 provides an overview of the number of red flags that were required for each symptoms and the complexity of the algorithms.

**Table 1:** Number of decision nodes and red flag questions in each symptom algorithm

Symptom	Decisional Nodes	Red Flag Questions
Nausea & Vomiting	54	6
Pain	257	2^a^
Severe Pain	125	
Moderate Pain	122	
Constipation	51	4

^a^Red flag questions are the same for both the moderate and severe pain pathways

### Phase 2: Usability testing

#### Participant sample

One-hundred-and-three patients were screened to identify the 24 patients and caregivers for study inclusion (Table 2). Twenty-six patients were deemed ineligible by their clinicians due to the incapacitating nature of their condition and 14 did not meet other eligibility criteria or could not be contacted. Seventeen potential subjects declined participation; 22 indicated interest but were unable to attend scheduled sessions or did not respond to scheduling requests. Participating patients and caregivers were predominately Caucasian, almost evenly split between men and women and most had some college level education.

**Table 2:** Cancer Patient and Caregiver Demographics (*N* = 24)

Characteristic	*n*	%
Role		
Patient	15	63
Caregiver	9	37
Gender		
Female	13	54
Age		
Median/Range	55	21–69
< 50	5	21
≥ 50	19	79
Race		
Caucasian	20	83
Black/African American	2	8
Other	2	8
Ethnicity		
Hispanic	4	17
Non-Hispanic	18	75
Did Not Report	2	8
Education		
High School or Less	3	12
Some College or More	21	88
Income		
$49,999 or Less	4	17
$50,000 or More	19	79
Did Not Report	1	4
Cancer Type (*n* = 15)		
Hematologic Malignancies	5	33
Solid Tumor Malignancies	10	67
Types of Solid Tumor		
Breast	1	7
Gastrointestinal	3	20
Genitourinary	1	7
Gynecologic	1	7
Head and Neck	1	7
Neuro-oncology	1	7
Thoracic	2	13
Internet Use to Obtain Health Information		
Never/Rarely	0	0
Sometimes	10	42
Often/Very Often	13	54
Missing	1	4

Forty-four clinicians were contacted to identify 13 who participated in focus groups and interviews (Table 3). Of the clinicians who did not participate, 20 did not respond, 2 declined, and 9 indicated interest but were unable to attend scheduled sessions. Clinician participants were predominately female and Caucasian.

**Table 3:** Oncology Clinician Demographics (*N* = 13)

Characteristic	*n*	%
Gender		
Female	11	85
Race		
Caucasian	11	84
Black/African American	1	8
Asian	1	8
Ethnicity		
Non-Hispanic	11	85
Did Not Report	2	15
Training		
Physicians	3	23
Nurse Practitioners	4	31
Physician Assistants	2	15
Registered Nurses	4	31
Cancer Specialty Area		
Hematologic Malignancies	4	31
Solid Tumor Malignancies	9	69
Types of Solid Tumor		
Gastrointestinal	2	15
Genitourinary	1	8
Neuro-oncology	1	8
Head and Neck	1	8
Thoracic	2	15
Radiation Oncology	1	8
General Practice	1	8
Prior Use of Patient-Focused Information Tools		
Never/Rarely	8	61
Sometimes/Often/Very Often	5	39

#### Focus groups and interviews

Patients and caregivers provided feedback about the visual appeal of the program, especially related to the color scheme, text density, and font size. Participant feedback sought to make the display inviting and easy to read. Some of the issues raised by clinicians who viewed the program were similar to issues identified by patients and their caregivers. For example, clinicians made suggestions about use of language, decreasing the density of text and providing graphic images to enhance understanding. For the most part, patients, caregivers and clinicians had similar comments to enhance the usability of the program. However, clinicians provided additional feedback about the content of the algorithms, and to ensure patient safety. They suggested adding questions at the beginning of the algorithms that would identify potentially dangerous symptoms that needed immediate attention and prompt patients to exit the program and call their clinicians. We also found that clinicians disagreed about what were best practices for self-management in pain management. Some clinicians felt that patients with pain levels of ≥ 9 should not self-manage and call their clinicians immediately, whereas others felt that it was acceptable to use the suggestions and then call their clinicians for refractory pain. Tables 4 and 5 provide an overview of comments provided by participants to improve the usability of the program.

**Table 4:** Results of Usability Testing for the SAMI-Self-Care CDS Program: Patient Perspectives

Usability Testing Themes	CDS Tool Content*	CDS Tool Component**	Examples
Visual appeal	Comments about introductory pages that had a lot of content	Visual appeal and design	“Some pages seem overwhelming.”
Understanding of terminology	Pain severity question	Written content and terminology	“What does ‘bearable pain’ mean?”
	Medication questions for all symptoms	Written content and terminology	“What does ‘able and willing’ [to take a medication] mean?”
	Nausea and vomiting question	Written content and terminology	“Position change- does that mean when I lift my head up?”
	Medication questions	Written content and terminology	“Unclear about the word ‘dose’ in the questions.”
	Medication question	Written content and terminology	“Did you take the dose you were due for? Does that mean the dose time already passed or the next dose I am due for?”
	Pain quality question	Written content and terminology	“Define what type of pain you are referring too”, is it “pokey pain, electrical current, shock pains burning pains, etc.”
	Pain quality question	Written content and terminology	“I wouldn’t have categorized numbness as pain...I’m glad it’s there.”
	Pain severity question	Written content and terminology	“I like ‘faces’ as part of the pain scale. They make the pain measure more clear.”
	Constipation definition	Written content and terminology	“Definitions were too wordy, for example, constipation definition had too much information.”
	Pain medication list	Written content and terminology	“I have trouble understanding meaning or relevance to words such as Morphine or Opioids.”
	Constipation medication list	Written content and terminology	“Is Senna tea the same as Senna medication?”
	Nausea and vomiting red flag safety questions	Written content and terminology	“What are two glasses of water per day?”
	All symptom medication questions	Written content and terminology	“Need to add a time frame to the question: ‘Did you take your medication?’”
	General content related to introduction of program and definition of all symptoms	Written content and terminology	Simplicity of terminology required for some patients with little medical sophistication makes clinical concepts difficult to communicate and can be tedious for more medically sophisticated patients.
	Nausea and vomiting questions	Written content and terminology	“Why are you asking me about acid reflux and then position change? Are they related?”
	Nausea and vomiting red flag safety questions	Written content and terminology	“Why is bone marrow transplant question asked?”
	Pain red flag safety questions	Written content and terminology	Reason for why some questions are asked is not understood, e.g., “Not everyone has back pain.”
	Nausea and vomiting questions	Written content and terminology	“Some patients may be getting some agents that aren’t considered chemotherapy but the patient thinks they are getting chemo.”
	Constipation questions and medication lists	Written content and terminology	“I didn’t know that morphine and opioids can cause constipation.”
	Pain and nausea and vomiting medication questions	Written content and terminology	“Don’t you want to know exact time and date of [a medication] dose?”
	Pain questions and medication list	Written content and terminology	Word “narcotic” brought up negative feelings it was “a scary word.”
	Pain question and medication list	Written content and terminology	“I know narcotic is bad for you.”
	General comment from bilingual participants	Written content and terminology	“Is this available in Spanish?”
Format and navigation	Pain and nausea and vomiting medication questions	Format and navigation	“Can we input all the medications we are taking into the system?”
	All symptom assessment questions	Format and navigation Suggestions to improve	“Add checkboxes to make this [the entry of symptoms] easier.”
	All symptom questions	Written content and terminology	“Create an option of ‘I don’t know.”
	General comment about iPad functionality	Format and navigation	“Are there instructions for those who are not computer users to know how to use this function?” (Referring to functionality of hovering over a definition for more information.)
	General comment on iPad	Format and navigation	“Use ‘back’ instead of ‘previous.’”
Wording of self-management suggestions	General comment for instructions for call clinicians on report	Written content and terminology	“I don’t want to bother my care team.”
	General comment for instructions to call clinicians on report	Written content and terminology	“When should I contact my care team?”
	General comment for instructions to call clinicians on report	Written content and terminology	“What should I tell my care team?”
Other			
Patient safety	Pain report suggestions	Written content for report	“Is it safe for me to take this medication?”
	Nausea and vomiting red flag safety questions	Written content for safety questions	“Are we allowed to drink 2 large glasses a liquid per day? Shouldn’t we ask the doctor first?”
	Constipation report suggestions	Written content for suggestions	“Is it safe for me to initiate the proposed intervention?”
Resources	Constipation suggestions	Other concerns	“Do I have suggested medications in my home?”
	General comment about iPad	Format and navigation	“Some people will lack the technology to access the system.”

* CDS tool content refers to what aspect of the CDS tool that the comment sought to improve (i.e. medication vs. pain severity question)

** CDS tool component refers to what aspect of the CDS tool that the component that the comment sought to improve (i.e. written content vs. visual appeal)

**Table 5:** Results of Usability Testing for the SAMI-Self-Care CDS Program: Clinician Perspectives

Usability Testing Themes	CDS Tool Context*	CDS Tool Component**	Examples
Visual appeal	General comment related to the look and feel of the system	Format	“Use larger fonts and colors as a way to distinguish instructions from question.”
	Nausea and vomiting	Format	“Give a visual description of what a 16-oz container might look like, e.g., a Poland spring water bottle.”
Understanding of terminology	Pain severity question	Written content and preference for terminology	Disapproval of wording, “bearable pain.”
	Nausea and vomiting question	Written content	“Clearly indicate what issue is being evaluated, e.g., [for] position change, are we asking about getting up quickly or vertigo?”
	Pain question	Written content	“Add timeframes, e.g., did taking the pain mediation offer you relief after 30 min?”
	Nausea and vomiting	Format	“Add graphics such as [a picture of] fire in the esophagus, which doesn’t need a definition.”
	All symptoms	Written content and format	“Medication lists might be overwhelming for some patients.”
	Pain	Written content	“Offer educational explanation such as risk factors regarding why the patients shouldn’t take certain medication, e.g., for ibuprofen explain why stomach protection is needed for those 65 or older.”
Format and navigation	Comment about introduction and orientation to the program	Format	“Select a symptom that is bothering the patients the most, and then come back to evaluate other symptoms.”
	Nausea and vomiting question	Written content and algorithms. This related to the issue that chemotherapy induced nausea may be more common than position-induced nausea	“Prioritize question order based on frequency of issues experienced by the patients to reduce number of questions patients have to answer and to avoid patients having to answer questions to symptoms majority might not experience.”
	Comment to improve the look and feel of the program	Format and navigation	“Try to reduce the number of clicks needed to move the system forward, e.g., they shouldn’t have to select the symptom and press next to move forward.”
	Provide an introduction to questions so patients will know what to expect and why	Format and navigation	“Tell patients upfront the different symptoms or medications the program will ask about.”
	General comment related to sequencing of questions	Format and navigation	“Add skip patterns for those who might have used the system before.”
	General comment about sequence of questions	Written content	“Work on lessening redundancy of the questions.”
Wording of self-management suggestions	Symptom reports	Written content	“Be clear with instructions regarding communication w/ clinicians.”
	Symptom reports for red flag questions.	Written content	“Clearly indicate to the patient to call now, so they do not mistakenly think the report has been automatically sent to their clinician and that someone will follow up.”
	Symptom reports	Written content	“Educate the patient on how to use the paging service.”
	Symptom reports	Written content	“Don’t put ‘during normal business hours’ because it sounds like we’re telling patients to stop bothering us.”
	Symptom reports	Written content	“List phone number of clinician on the report or a paging service for after hours.”
Other			
Patient safety	All symptoms red flag questions developed for safety	Algorithm content	Identify all red flag/emergency issues.
	Pain red flag safety questions	Algorithm content	“Ask about new or severe pain not just one [or the other] and, [a] ‘yes’ [response] should mean call your doctor right away.”
	General comment about reports that are generated for self-management	Written content	Clinicians worried that they may not be informed about patient problems.
	All symptoms red flag questions added	Algorithm content	Clinicians concerned they may miss or overlook critical situations.
	General comment about the report	Written content	“Include notification to patient that they should always call provider with questions.”
	General comment	Written content	“Wouldn’t want the patient to use the program instead of getting care.”
Resources	General concern about use of iPad	Format	Concern that some patients will not have access to computers.
Best care practices	Pain self-management for severe pain	Written content about when to call their clinicians	Lack of consensus among clinicians regarding clinical best practices.
	Nausea and vomiting acid reflux	Algorithm content	Some providers recommend medication like TUMS, but GI doctors may avoid it because it creates acid.
	Pain and nausea and vomiting	Written content wanted more comprehensive lists for medications	Recommended some medications be added on to lists [of medications already included].
	Pain medication question	Written content	“Change dosing criteria for long acting to 8–12 h.”
	Pain medication lists	Written content	“Certain medications on the list not used across the board causing worry, e.g., fentanyl or tapentadol.”

* CDS tool content refers to what aspect of the CDS tool that the comment sought to improve (i.e. medication vs. pain severity question)

** CDS tool component refers to what aspect of the CDS tool that the component that the comment sought to improve (i.e. written content vs. visual appeal)

### Acceptability surveys

Participants completed surveys regarding the acceptability of SAMI-Self-Care for “nausea/vomiting” and “pain” after each round of system development and viewing the simulated interface. Nine patients and their caregivers completed surveys, four assessing pain and five assessing nausea and vomiting. Two rounds of assessment for usability and acceptability were conducted with patients to reach or surpass the predetermined threshold for acceptability. In general, patients found the system easy to understand and helpful for self-management.

Thirteen clinicians completed surveys, nine assessed pain and three assessed nausea and vomiting, and one assessed constipation. Two rounds of assessment for usability and acceptability were conducted with clinicians. The simulated program for nausea/vomiting and constipation reached and surpassed the threshold for acceptability. However, the helpfulness of suggestions for the pain algorithm was scored lower than our target threshold of 4.0. For the most part, clinicians also viewed the system for pain as usable and helpful. Subsequent discussions revealed that some clinicians felt that patients with a pain score ≥ 9 should contact their clinicians and not pursue self-management. The algorithm was subsequently changed to reflect that patients with severe pain, should contact their clinicians, but time limitations prevented the modified algorithm from being reevaluated.

### Phase 3: Design Objectives and Barriers to Patient-centered CDS

#### Design objectives

From patient, caregiver, and stakeholder feedback, we identified five design objectives that were relevant for the development of algorithm-based patient-centered CDS, which included: ensure patient safety, communicate clinical concepts effectively, promote communication with clinicians, support patient activation, and facilitate navigation (see Table 6). This table includes information about the design objective, specific stakeholder barriers related to this objective, solutions that were identified to address the barrier and changes that were made in the user interface. A discussion surrounding each of these design objectives is found in the following section followed by some barriers to patient-centered CDS that emerged from our findings.

**Table 6:** Design Objectives for Development of Patient-Centered CDS

Design Principle	Design Principle Details	Examples of Solutions	Change in User Interface
1 Ensure Patient Safety	1a Build algorithm content based on established clinical guidelines	Use of the NCCN guidelines for cancer pain management to guide algorithm content [63]	Based on published best practices, evidence-based content used for developing symptom management algorithms
			Iterative review process of algorithm content and recommendations by multidisciplinary expert panel members
	1b Identify at the beginning of a session potentially serious conditions for which continued use of algorithm could be harmful or life threatening	Additional characteristics of symptoms that suggest potentially dangerous or life threatening conditions identified. e.g., “in pain algorithm, besides enquiring about new or increased pain, adding a question that asksabout cramping or squeezing in chest or stomach”.	Questions added that identify severity and trigger “call now” advice.
			CDS updated for immediate exit and to contact clinician if red flag was triggered
		Distinguished nuances between pain symptoms, (e.g. new pain (e.g. fracture) and chronic pain)	Any time red flag is triggered, patient provided with specific suggestions on the screen.
		Disclaimer needed to ensure safety (e.g. “In case of emergency, call your doctor or 911 immediately. Do not use this program for medical emergencies.”)	Warning placed on the welcome page of the program.
			Bold font used as a way to capture patients’ attention.
		There should be gradation of severity indicating what issues should the patient address first	Visual cues added to the report to help prioritize self-management strategies
			Colors (red-orange-green) and fonts used to ensure patient reviewed specific aspect of the report. e.g. call clinician now
			Report provided at the end can be viewed on the screen or as a printed report
	1c Inquire about appropriateness of recommendations prior to offering advice	Provide guidance on why particular intervention should not be implemented (e.g. taking ibuprofen)	Content modified to provide reasons why a particular intervention would not be permissible (e.g. stomach ulcers)
		Provide language to ensure any questions are directed to care team at all times	Report content updated to contact clinician if uncertainty about concerns on implementing recommendations
2 Communicate Clinical Concepts Effectively	2a Test word selection with intended end-users	Cognitive testing of terms and its interpretation	Modified wording utilized in assessment and recommendations to improve understanding of concepts
	2b Develop explicit, detailed questions	Remove ambiguity of decision points	Added specificity of timeframes to questions to improve meaning (, e.g., “Did taking short acting pain medication give you relief from your pain within 30 min of taking it?”)
			Reference specific medications and dosages as appropriate
			Designed explicit decisions points to enable machine processing
	2c Enhance communication with graphics, especially for clinical concepts	Improve system use by reducing content	Added “faces” and word anchors as part of the pain scale
			Created content at a 5th grade reading level
			Inserted images to re-enforce concepts (e.g., stop sign for emergency, picture to show acid reflux)
	2d Provide lists to enable patient to identify specific items such as medications	Utilization of system could be improved by equipping patients with necessary information	Provided lists of most common medications in defined classes in a designated area of the screen for lookup as needed
			Included generic and brand names of medications for ease of recognition
	2e Provide educational information to promote understanding	Using CDS as a way to reinforce and provide education on why certain questions are being asked	Educational content added in final summary reports customized to their symptoms
			Provided rationale of why certain questions were asked and promoted understanding
	2f Enhance readability with font style, font size, content density, selective highlighting of words	Improve utility by improving layout of content	Used large and “heavier” font size to make text more visible
			Reduced text density
			Used a plain white background
			Provided bolding to emphasize words
3 Promote Communication with Clinicians	3a Provide explicit instructions for patients regarding contacting clinicians about their concerns	Urgency of establishing clinical contact based on severity of the symptom needed(e.g. call right away vs. waiting 24 h)	Additional features added to generate report immediately on screen if patient triggered any of the emergency “red flag” questions and highlighted the importance of calling clinician NOW.
		Post assessment report that provides guidance on what should be done and when.	Immediate instructions provided to the patient, on calling clinician, onscreen of the program and not just within the report.
			Added explicit language on what patients should say when calling clinician.
		Initial reports lost the message about the importance of communicating with the clinician	Report restructured to reinforce importance of contacting clinicians and keeping them informed of regimen changes. E.g. tell your doctor or nurse you are taking 200 mg of ibuprofen as needed.
		Clearly communicate recommendations	Report modified into sections of: do now, do next and more suggestions, to help streamline and prioritize suggestions for what the patient can do and when
		Lack of specification of which symptoms are available for assessment at beginning of the program	3 symptoms patients can choose in current system listed at the beginning of the program.
			Patients advised to contact clinicians if experiencing symptoms not addressed by the system.
	3b Encourage patients to notify their clinical care team about interventions that they have followed	Reinforce the importance of notifying clinicians about any interventions that have been initiated within the recommendations	Provided instructions about what patients should specifically tell their clinicians about interventions
4 Support Patient Activation	4a Determine what resources are available to the patient	Improve efficiency of the system and utilization by modifying question based on what patients have available to them	Added questions to determine what interventions had already been prescribed
			Inquired if a prescription was already available for a recommended medication as a way to align with current therapy of the patient’s care team
	4b Identify health beliefs that may impact interpretation of content and modify content accordingly	Modifying how content is framed	Content modified conveying meaning acceptable by patients. (e.g. pain medication vs. narcotics)
	4c Determine what patients are willing to do prior to making recommendations	Improving look and feel of the system that quickly provides information and allows the patient to take an active role in their care	Provided explicit, detailed instructions that include dosage amounts, frequencies, medication list and lifestyle suggestions
			Prioritized display in patient report to quickly and easily inform the patient on what they should do next
	4d Provide explicit, detailed, actionable instructions to the extent possible		Inquired about what patients were willing to do prior to recommending an intervention, e.g., use of enemas for constipation
	4e Personalize content, e.g., used possessive pronouns such as “my” or “your” where appropriate	Create an opportunity relate to the patient and provide self-management techniques	Changed the text to make it personable and user friendly, e.g., used possessive pronouns such as “my” or “your” where appropriate
5 Facilitate Navigation and Use	5a Designate consistent presentation areas on screen for repeated display of a specific type of information	Make it easy for patients to find information within the site	Posted medical terms with definitions in a specific area on the screen so end user can easily and quickly access information as needed
			Avoided “pop-ups” because they felt to be interruptive and harder to navigate for a limited computer proficient user
	5b Provide comprehensive set of selection options	Ensure all possible decision points are covered	Guidelines and best practices used for comprehensive coverage to ensure all possible selections covered for every decision node
	5c Streamline data entry	Improve flow and provide feedback quickly	Introduced check boxes to cut down on number of questions required to determine what advice to provide and improved efficiency
	5d Optimize workflow through questions	Inquiring about symptom characteristics at the beginning of the algorithm	Enabled selection of an item on a page to advance to the next page as appropriate
			Directed patients to highly specific interventions
	5e Optimize workflow through questions	Inquiring about symptom characteristics at the beginning of the algorithm	Facilitated patients starting at the appropriate place in the algorithm by inquiring what interventions have already been attempted
			Introduced check boxes to reduce number of question and reduce redundancy
	5f Track progress for patient	Promote efficient workflow	Added progress bar showing numeric value, not just graphic representing progress
			Included “Go Back” function to allow patient to modify earlier responses
	5g Accommodate patient changes and pauses		Offered multiple ways to start over such as: “Back to Start button” as well as tabs with symptom names
			Included “Take a Break” button to allow patient to pause the program and come back to it again
	5h Provide context for all interactions so that patient recognizes where he/she is within an algorithm		Added tabs as a way to indicate to the patient which algorithm they were in
			Provided headers to supply context for each page anchoring the patient on where they are in a given algorithm
	5i Ensure completeness and uniqueness of pathways through algorithm		Provided brief overview of different topics that were covered to orient patients at the beginning of a session
			Re-enforced context and inter-relatedness of questions by showing question and answer from the previous page
			Ensured that questions allow for a single non-redundant, unique pathway for all possible scenarios
			Ensured that every pathway led to advice
	5j Create tools that will function across multiple platforms	Assessed target patient population to determine that 85% of patients had access or knew how to obtain access to computers or smart phones	Created CDS tool design to function on Web, smart phone, or iPad

#### Ensure patient safety

To ensure that the use of SAMI-Self-Care would not overlook a life-threatening condition, we identified a set of screening questions for each symptom that sought to direct patients with potentially dangerous conditions to stop using the algorithm and seek contact with their clinicians. To ensure that the advice provided through SAMI-Self-Care was sound, we derived algorithm logic and recommendations from evidence-based clinical guidelines. When appropriate, we also inquired if a recommended therapy had already been suggested by the patient’s clinician. If the therapy had not been suggested, we encouraged the patients to check with their clinician to request approval before initiating a new medication.

#### Communicate clinical concepts effectively

In order to communicate clinical concepts effectively, we conducted cognitive testing of content with patients to ensure an adequate level of understanding, and we added graphics to support concepts in the text when appropriate. We also designated a section of the display for explanations or lists so that patients who needed more information could easily access it. Formative testing of questions through the cognitive interviewing process helped us to recognize that we needed to make questions as explicit and detailed as possible so patients were not left to speculate about how to respond. Finally, we used established interface development practices for screen appearance, layout, and text fonts to enhance readability [45].

#### Promote communication with clinicians

To encourage communication with patients’ clinicians, we included explicit suggestions regarding when to contact their clinicians and a script for what to say to them. We also advised patients to notify their clinicians within a specified time frame about interventions they may have followed from SAMI-Self-Care.

#### Support patient activation

We sought to engage patients by providing personalized and actionable instructions. As appropriate, we asked patients about resource availability and their willingness to follow interventions prior to making recommendations. Through the educational display section, we provided content to help patients understand why certain questions were being asked and why specific recommendations were provided. Helpful features included suggestions for pharmacologic and non-pharmacologic therapies.

#### Facilitate navigation and use

To expedite SAMI-Self-Care use we identified methods to optimize traversal of the algorithms and minimize the burden of data entry (see Table 6). We included a progress bar for patients to know their position in the process, and we added capabilities to change responses or to pause a session. We also added display content to enable a patient to recognize the context of every interaction through tabs illustrating the symptom being addressed and text clarifying the context of each question. Finally, we ensured that all pathways could be traversed, were unique and not redundant.

### Barriers that need to be addressed to promote patient-centered CDS

Barriers to promote patient-centered CDS regarding symptom self-management that emerged appeared to be consistent with the Chronic Care Model so the themes that emerged were reorganized around the components of this model which included: patient safety, cultural competency, care coordination, resource availability, and acceptance of technology.

Barriers pertaining to cultural competency were the most common and included challenges related to unfamiliar terminology, lack of reference to a clinical framework, and unusual health beliefs**.** Although many of the concerns identified by patients were shared by clinicians, a few items were unique to patients and their caregivers, including expressing uncertainty about some self-management suggestions and whether the actions would be acceptable to their clinicians, expressing negative health beliefs associated with the use of narcotic medications, and not having the suggested medications available at home. There also were concerns about communication with their clinicians, availability of technology in the community, and use of the technology.

## Discussion

We developed a simulated algorithm-based CDS program to support symptom self-management among adults with cancer and their caregivers. Our CDS program is more complex than other previously developed programs and provides patents with specific information, tailored to their situation regarding when to call their clinician, what to tell them, and how to self-manage their symptoms. In order to accomplish this task, we have many more decisional nodes than previous patient-centered CDS systems [12, 22]. Acceptability testing results among patients were favorable, suggesting that patients found the SAMI-Self Care prototype a satisfactory approach to for symptom self-management. Providing CDS directly to patients may be a valuable tool. Future studies should explore the best modalities for providing access to these tools including the Web, tablet computers, or mobile phones [46]. We anticipate that the SAMI-Self-Care tool could be implemented on a Web-based platform that could be accessible across computers, tablets and smartphones. A link to this content could be inserted in the homepage of a patient’s oncology treatment group or sent out directly to patients through electronic mail.

We noted that the acceptability results from clinicians for pain were less favorable than for nausea/vomiting and constipation, and were below our desired threshold of 4.0 for helpfulness of the report and suggestions. This result required obtaining a larger sample size to gather responses about usability testing as compared to other symptoms. In spite of our efforts to adhere to pain guidelines, [47] some clinicians were reluctant to support self-management because they wanted to remain aware of their patients’ symptoms and to be directly involved in management. This finding is interesting as implementation of pain guidelines has been a challenge. Evidence suggests that pain management practices have not changed over the last twenty years and many patients with cancer continue to experience severe uncontrolled pain [48, 49]. Further study is needed to understand clinician attitudes toward pain management and barriers to encouraging patient self-care management. One concern that clinicians expressed is that they wanted to be aware of patients who had severe levels of pain. The addition of a system that alerts clinicians when patient symptoms pass a predetermined level of severity would address this concern. Cleeland and colleagues [13] found that automated symptom severity alerts after surgery for lung cancer reduced symptom distress. In a recent study that examined clinician preferences for CDS, the use of an alerting system for increased symptom severity was identified as a desirable component [50].

Our design objectives were specifically developed in this study to support development of effective patient-focused CDS. To date, CDS design objectives have focused on use by clinicians. Thus, even though our CDS system was designed for patients, we sought to include features known to be associated with effective CDS systems for clinicians [35, 36, 51]. The system features identified in our study that were similar to clinician CDS included: graphics to enhance understanding of content; explicit, actionable recommendations provided at the point of decision-making; presentation of advice that cultivated trust by providing an explanation of medical logic if needed [36]. In addition, similar to Bates and colleagues, [35] our Design Objectives focused on streamlining the user interaction by decreasing the number of questions needed to inform the algorithm, targeting anticipated needs with advice in real-time, providing supplemental information as needed but without interruption, and relying on a user-centered iterative design [43]. Ozkaynak and colleagues [52] noted that an important difference between patient and clinician design for CDS is integrating technology into the user’s work flow. Designing CDS around clinician work flow is much easier than for patient work flow as clinicians function within a health system, whereas work flow for patients spans multiple health care settings and includes home, routines of daily living and communication with family, clinicians and the health care system [52–55]. The CDS program that was evaluated in this study attempts to bridge the gap between care delivered in health care settings and self-care in the home. There is a need for applications that improve outcomes across settings and patient populations [56]. Future studies are needed to assess the impact of this CDS program on patient outcomes.

Through this process, we identified patient barriers to use of CDS and clinicians’ concerns about patients using CDS for self-management. From these barriers and concerns, we derived Design Objectives for CDS for symptom self-management, which included ensuring patient safety, communicating clinical concepts effectively, promoting communication with clinicians, supporting patient activation and facilitating navigation and use. These objectives may be useful to inform the design of CDS systems for symptom self-management of other conditions.

The barriers we identified for CDS for cancer symptom self-management had similarities and differences to barriers associated with self-management in patients with non-malignant conditions [57, 58]. Similar barriers included lack of knowledge, poor communication between patients and clinicians, and logistical issues in obtaining care. However, in contrast to the patients who did not have cancer, the cancer patients did not report barriers related to physical limitations, financial constraints, a need for social and emotional support, or challenges adhering to treatment [57, 58]. Other differences in concerns that we noted between patients with cancer and those with non-malignant conditions were that cancer patients and their caregivers expressed concerns related to ensuring patient safety and negative health beliefs about use of narcotic pain medications. Shumacher and colleagues [59, 60] examined pain management processes among cancer patients and their caregivers and found similar concerns related to ensuring patient safety, especially in the context of narcotic pain medications. Patients and their caregivers had little interaction with clinicians in the home setting and had to master complex tasks related to taking their medications and reported that they felt that they had to be the final safety check.

### Limitations

The sample for this study was drawn from one institution using purposive sampling so it is not representative of all cancer patients. All of the patients and their caregivers in this study indicated that they used the Internet for seeking health information at least sometimes. This rate is higher than the 72% of Internet users found in the 2014 Pew Research survey [61]. Our rates for Internet usage may be higher as the majority of our sample had greater than a high school education. Factors associated with lower access of the Internet are older age, lower education and lower income [62]. Another limitation is that the majority of the sample was Caucasian and had higher levels of education. Further testing of this approach in a more diverse group of patients with cancer is warranted. Although the use of a simulated model provided a practical and economical approach to iterative development of the CDS tool, this approach did not allow for real time navigation through the algorithms for patients. This would be the next step in the development process.

## Conclusions

The boundaries of health care are expanding and patients and their caregivers often have to self-manage complex cancer care at home. Thus, patient-centered decision support that meets this need is important and timely. Our system provides tailored information that informs patients when to call their clinicians, provides a script about what to tell tem about their symptom and specific suggestions about how the self manage their symptoms at home. Patients and their caregivers rated SAMI-Self-Care as highly acceptable and found the recommendations helpful. The patient-centered CDS design objectives we derived for cancer symptom self-management may be applicable for the self-management of other conditions.

## Abbreviations

CDS: Clinical decision support
SAMI-Self-Care: Symptom Assessment and Management Intervention - Self Care
